# Non-Invasive Determination of Left Ventricular Workload in Patients with Aortic Stenosis Using Magnetic Resonance Imaging and Doppler Echocardiography

**DOI:** 10.1371/journal.pone.0086793

**Published:** 2014-01-28

**Authors:** Zahra Keshavarz-Motamed, Julio Garcia, Emmanuel Gaillard, Romain Capoulade, Florent Le Ven, Guy Cloutier, Lyes Kadem, Philippe Pibarot

**Affiliations:** 1 Laboratory of Biorheology and Medical Ultrasonics, University of Montreal Hospital Research Center (CRCHUM), Montréal, Québec, Canada; 2 Laboratory of Cardiovascular Fluid Dynamics, Mechanical and Industrial Engineering, Concordia University, Montréal, Québec, Canada; 3 Department of Radiology, Northwestern University, Chicago, Illinois, United States of America; 4 Québec Heart and Lung Institute, Laval University, Québec, Québec, Canada; 5 Department of Radiology, Radio-Oncology and Nuclear Medicine and Institute of Biomedical Engineering, University of Montreal, Montréal, Québec, Canada; Scuola Superiore Sant’Anna, Italy

## Abstract

Early detection and accurate estimation of aortic stenosis (AS) severity are the most important predictors of successful long-term outcomes in patients. Current clinical parameters used for evaluation of the AS severity have several limitations including flow dependency. Estimation of AS severity is specifically challenging in patients with low-flow and low transvalvular pressure gradient conditions. A proper diagnosis in these patients needs a comprehensive evaluation of the left ventricle (LV) hemodynamic loads. This study has two objectives: (1) developing a lumped-parameter model to describe the ventricular-valvular-arterial interaction and to estimate the LV stroke work (SW); (2) introducing and validating a new index, the normalized stroke work (N-SW), to assess the global hemodynamic load imposed on the LV. N-SW represents the global hemodynamic load that the LV faces for each unit volume of blood ejected. The model uses a limited number of parameters which all can be measured non-invasively using current clinical imaging modalities. The model was first validated by comparing its calculated flow waveforms with the ones measured using Cardiovascular Magnetic Resonance (CMR) in 49 patients and 8 controls. A very good correlation and concordance were found throughout the cycle (median root mean square: 12.21 mL/s) and between the peak values (r = 0.98; SEE = 0.001, p<0.001). The model was then used to determine SW using the parameters measured with transthoracic Doppler-echocardiography (TTE) and CMR. N-SW showed very good correlations with a previously-validated index of global hemodynamic load, the valvular arterial impedance (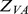), using data from both imaging modalities (TTE: r = 0.82, SEE = 0.01, p<0.001; CMR: r = 0.74, SEE = 0.01, p<0.001). Furthermore, unlike , N-SW was almost independent from variations in the flow rate. This study suggests that considering N-SW may provide incremental diagnostic and prognostic information, beyond what standard indices of stenosis severity and provide, particularly in patients with low LV outflow.

## Introduction

Aortic stenosis (AS) is a complex “systemic” disease. There are compelling epidemiological and histopathological data suggesting that “degenerative” calcified AS is, in fact, an active and multifaceted disease that involves atherosclerotic-like and elastocalcinosis-like processes [1], [2], [3].

The degenerative process is not limited to the aortic valve; it involves the vascular system distal to the valve as well, which in turn can further contribute to deteriorating of left ventricle (LV) function [4].

Moreover, in patients with severe AS, “paradoxical” low flow (stroke volume index (SVi) <35 mL/m^2^) and consequently low transvalvular pressure gradients (<40 mmHg) despite the presence of preserved LV ejection fractions (>50%), is a challenging clinical entity [5], [6], [7]. This mode of presentation of severe AS is relatively frequent (up to 35% of cases) and has been shown to be associated with a more advanced stage of the disease [5], [6], [7]. Yet, a majority of these patients do not undergo surgery due to inadequate diagnosis. In these patients, the presence of low pressure gradient in conjunction with a normal LV ejection fraction may easily lead to an underestimation of AS severity [5], [6], [7]. Prognosis in such patients is usually poor (survival rates <50% at 3-year follow-up) if treated medically and operative risk is high (up to 33%) if treated surgically [5], [8]. Early detection and accurate estimation of AS severity are therefore of primary importance. For proper diagnosis of these patients, a comprehensive evaluation of LV hemodynamic loads is crucial. Such a comprehensive evaluation helps to identify needs for closer follow-ups, and to improve risk stratification and clinical decision making [8].

Recently, Briand et al. (2005) proposed the valvulo-arterial impedance (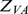) (equation 8) which represents the valvular and arterial factors that oppose ventricular ejection by absorbing the mechanical energy developed by the LV. This index has been shown to be a strong predictor of clinical outcomes in asymptomatic AS patients and is superior to the standard indices of AS severity in predicting LV dysfunctions [9], [10]. However, 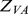 is flow-dependent and therefore has limited use specifically in AS patients with low flow and low pressure gradient conditions [8].

The LV stroke work has been shown to be effective in characterizing the LV loads and consequently in characterizing patient’s outcome and in assessing the inotropic state in patients with AS [11], [12], [13]. LV stroke work is the energy that the ventricle delivers to the blood at ejection, and potential energy, necessary to overcome the viscoelastic properties of the myocardium itself. Therefore, A method that would allow simple, non-invasive, and accurate estimation of the LV stroke work would contribute towards improving the clinical management of patients with AS and especially the ones with paradoxical low flow. However, estimation of this parameter requires the knowledge of instantaneous LV pressure and volume. Cardiac catheterization with conductance catheters can provide this information but this method is invasive, expensive and may cause cerebral embolisms [14]. A non-invasive alternative to estimate LV stroke work is to model the cardiovascular system using a lumped parameter model based on non-invasive recorded inputs.

The first objective of this study was therefore to develop and to introduce a simple lumped parameter model, solely based on non-invasive parameters, to describe the ventricular-valvular-arterial coupling and to investigate the impact of AS and vascular diseases on LV workload. The second objective was to introduce and validate a new index easily measurable in the clinical setting, *i.e.,* the normalized LV stroke work (N-SW). This index in J/mL, representing the energy required to eject 1 mL of blood through the valvulo-arterial system, is calculated by dividing the estimated LV stroke work by the stroke volume (SV).

## Methods

### Lumped Parameter Modeling

**Figure 1:**
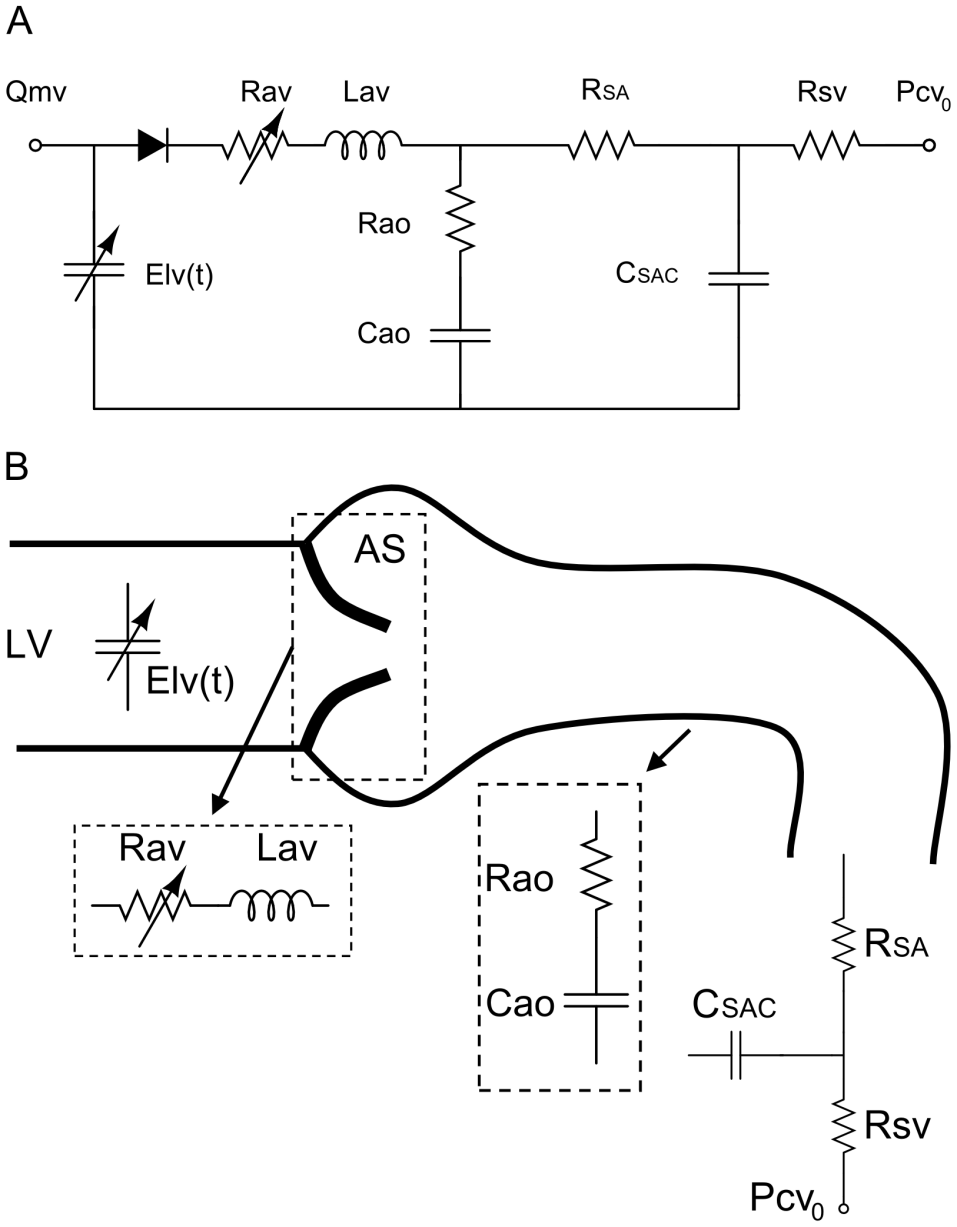
Schematic diagrams. (a) electrical representation, (b) schematic representation of the lumped parameter model used to simulate left-sided heart in presence of aortic stenosis and/or systemic arterial hypertension. LV: left ventricle, AS: aortic stenosis, E_lv_(t): normalized time-varying elastance (please see Table 1 for all other parameters used in the lumped parameter model).

A schematic diagram of the lumped parameter model is presented in Fig. 1 [15]. This model includes three different sub-models: 1) LV model; 2) AS model; 3) systemic circulation model. The effect of AS on LV workload was investigated under several numerical conditions using datasets obtained non-invasively by transthoracic echocardiography (TTE) and cardiovascular magnetic resonance (CMR) in healthy subjects and in AS patients. All other parameters used in the lumped parameter model are listed in Table 1.

**Table 1:** Summarized cardiovascular parameters used to simulate all cases.

Description	Abbreviation	Value
**Aortic valve parameters**		
Effective orifice area	EOA	From TTE and CMR data
Aortic cross sectional area	A_ao_	From TTE and CMR data
Valvular energy loss coefficient	E_L_Co	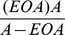
Variable aortic valve resistance	R_av_	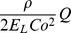
Aortic valve inductance	L_av_	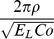
**Systematic circulation parameters**		
Aortic resistance	R_ao_	0.05 mmHg.s.ml^−1^
Aortic compliance	C_ao_	0.5 ml/mmHg
Systemic vein resistance	R_SV_	0.05 mmHg.s.ml^−1^
Systemic arteries and veins compliance	C_SAC_	Initial value: 2 ml/mmHgAdjust for each degree of hypertension
systemic arteries resistance(including arteries, arterioles and capillaries)	R_SA_	Initial value: 0.8 mmHg.s.ml^−1^Adjust according to the obtained total systemic resistance
**Output condition**		
Central venous pressure	P_CV0_	4 mmHg
**Input condition**		
Mitral valve mean flow rate	Q_mv_	
**Other**		
Constant blood density		1050 kg/m^3^
Cardiac output	CO	From TTE and CMR data
Heart rate	HR	From TTE and CMR data
Duration of cardiac cycle	T	From TTE and CMR data

#### Heart-arterial model

Heart function was described by time varying elastance as following
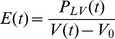
(1)where P_LV_(t), V(t) and V_0_ are the left ventricular pressure, the left ventricular volume and the unloaded volume [15], respectively. The amplitude of E(t) was normalized with respect to maximal elastance E_max_, i.e., the slope of the end-systolic pressure-volume relation, giving E_N_(t_N_) = E(t)/E_max_. Time then was normalized with respect to the time to attain peak elastance, T_Emax_ (t_N_ = t/T_Emax_). Normalized time-varying elastance curves E_N_(t_N_) have similar shapes in the normal human hearts with various inotropic situations or for diseased human hearts despite the existence of differences with regard to etiology of cardiovascular diseases [16], [17]. More details can be found elsewhere [15]

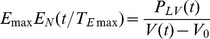
(2)


#### Modeling aortic stenosis

Aortic stenosis was modeled using the semi-analytical formulation for the net pressure gradient 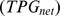 across the stenotic valve during LV ejection introduced by Garcia et al. [18]. This formulation expresses the instantaneous net pressure gradient across the stenotic valve (after pressure recovery) as a function of the instantaneous flow rate and the energy loss coefficient and links the LV pressure to the ascending aorta pressure 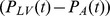


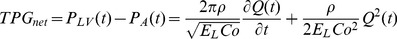
(3)and
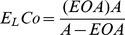
(4)where 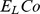, 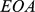, 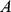, 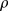 and 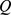 are the valvular energy loss coefficient, the effective orifice area, the aortic cross sectional area, the fluid density and the transvalvular flow rate, respectively.

#### Computational algorithm

The lumped model illustrated in figure 1 was analyzed numerically by creating and solving a system of ordinary differential equations in Matlab Simscape (MathWorks, Inc). Capabilities of this program were enhanced by adding additional codes to meet demands of cardiac circuit. Fourier series representation of experimental normalized elastance curve for human adults was used to generate a signal to be fed into the main program. Equation 3, representing the transvalvular pressure gradient across the aortic valve, was represented by an inductance and a variable resistor as depicted in figure 1. Simulation started at the onset of isovolumic contraction and the elastance signal drives the program by feeding elastance value related to each time step in the cycle to the equation 1. The left ventricle volume 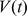 was calculated using the left ventricle pressure 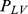 and elastance values by equation 1. The 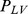 used at the beginning of calculation was the initial value assumed across the variable capacitor and was automatically adjusted later by the system of equations as solution advances. The left ventricle flow rate subsequently was calculated as the time derivative of the left ventricle volume. After few initial cycles, solution converged. A diode with very low on resistance and off conductance was used in the aortic valve to prevent backflow from the valve. Matlab’s “ode23t” trapezoidal rule variable-step solver was used to solve system of differential equations with initial time step of 0.1 milliseconds. The Convergence residual criterion was set to 10^−5^ and initial voltages and currents of capacitors and inductors set to zero.

#### Determining arterial compliance and peripheral resistance

The total systemic resistance was computed as the average brachial pressure over the cardiac output (assuming a negligible peripheral venous pressure (mean ∼5 mmHg) compared to aortic pressure (mean ∼100 mmHg)). This total systemic resistance represents the electrical equivalent resistance for all resistances in the current model. Because what the left ventricle faces is the total systemic resistance and not the individual resistances, for the sake of simplicity, we elected then to consider the aortic resistance, 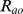, and systemic vein resistance, 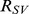, as constants and to adjust the systemic artery resistance, 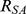, according to the obtained total systemic resistance.

Physiologically, arterial hypertension is determined by two factors [19]: (1) a reduction in the caliber of small arteries or arterioles with an ensuing increase in systemic vascular resistance and mean blood pressure, and (2) a reduction in the arterial compliance with a resulting increase in pulse pressure (systolic minus diastolic blood pressure). In this study, we fitted the predicted pulse pressure to the actual pulse pressure (known by arm cuff sphygmomanometer) by adjusting the systemic compliance (C_SAC_). Therefore, C_SAC_ adjustment was done by a simple trial and error for each degree of hypertension [20].

### *In vivo* Measurements

#### Ethics statement

This study was approved by the Ethics Committee of Research of Laval Hospital affiliated to Laval University. Informed consents were obtained from all patients.

#### Study population

Forty-nine patients with mild to severe AS (63% men, age 63±16 years) and eight healthy subjects (75% men, age 34±8 years were included in this study (Table 2). Exclusion criteria were: (1) age <21 years old. Patients with AS are often older than 21 years, also younger patients are treated as pediatric population according to the American Heart Association guideline; (2) LV ejection fraction <50%. SV measurements in patients with reduced LV ejection fraction may show inconsistencies; (3) moderate or severe mitral or aortic regurgitation. SV measurements in these patients may show inconsistencies; (4) poor TTE data quality and standard contra-indications to CMR data. To demonstrate the feasibility of our method, only patients with useable data were considered in our study. In 33% of AS patients, the valve morphology was bicuspid. Other measured characteristics of patients were: height (166±9 cm), weight (76±13 kg), waist circumference (95±11 cm), blood pressure (128±22 mmHg/71±11 mmHg), body surface area (1.82±0.23 m^2^), diabetes (16%) and metabolic syndrome (35%). The summary of performed measurements is presented in Table 2. All patients provided written informed consent under the supervision of the Institutional Review Board. The initial AS severity classification at study entry was based on TTE-derived effective orifice area (EOA): normal (EOA>2.0 cm^2^), mild (1.5<EOA≤2 cm^2^), moderate (1.0 cm^2^<EOA≤1.5 cm^2^) and severe (EOA≤1.0 cm^2^).

**Table 2:** Baseline characteristic data.

	Healthy Subjects	AS Patients
	(n = 8, mean ± SD)	(n = 49, mean ± SD)
**Patient description**		
Age (years)	34±8	63±16*
Sex (Men %)	75	63
Body surface area (m^2^)	1.93±0.26	1.82±0.19
**Left ventricle function and geometry**		
Left ventricle end-diastolic internal dimension (mm)	50±3	45±5
Interventricular septal thickness (mm)	9±1	12±2*
Left ventricle mass (g)	230±54	194±55
Left ventricle mass index (g/m^2.7^)	52±14	49±52
Left ventricle ejection fraction (%)	66±4	66±5
**Arterial hemodynamics**		
Systemic arterial compliance (mL.m^−2^.mmHg^−1^)	1.17±0.32	0.78±0.26*
Systemic vascular resistance (dyne.s.cm^−5^)	1335±284	1801±774
Systolic arterial pressure (mmHg)	116±10	128±22
Diastolic arterial pressure (mmHg)	77±5	71±11
Mean arterial pressure (mmHg)	90±7	90±13
Valvulo-arterial impedance (mmHg/mL/m^2^)	3.35±0.71	3.66±0.85
**Valve hemodynamics**		
Mean transvalvular gradient (mmHg)	5±1	21±11*
Effective orifice area (cm^2^)	2.67±0.47	1.31±0.59*
Aortic diameter (mm)	31±4	32±4
**Left ventricular stroke work**		
Stroke work (J)	0.85±0.2	1.45±0.75
Normalized stroke work (J/mL)	0.009±0.002	0.017±0.007

* p<0.05 with healthy.

#### Transthoracic Doppler-echocardiography (TTE)

TTE exams were performed and analyzed by two experienced echocardiographers, and conducted according to the American Society of Echocardiography guidelines [21]. They included:

- Valve hemodynamic parameters: the left ventricle outflow track (*LVOT*) diameter, *LVOT* flow velocity measured by pulsed-wave Doppler, the aortic transvalvular jet velocity measured by continuous-wave Doppler and valve effective orifice area (EOA) using the continuity equation as follow,
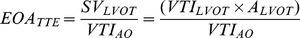
(5)where 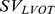, 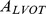 and 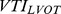 are the stroke volume measured in the LVOT, the cross-sectional area of the LVOT calculated assuming a circular shape (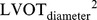
×0.785), and the velocity-time integral of the LVOT, respectively.
- Parameters of LV systolic function: the left ventricle ejection fraction (*LVEF*), Sa wave and left ventricle geometry [22];
- Parameters of LV diastolic function: Ea wave, *E/A* ratio, left ventricle geometry and mass indexed to height^2.7^ (*LVMi*) [23], [24];
- Vascular hemodynamic parameters: the systemic arterial compliance (*SAC*) and the systemic vascular resistance (*SVR*) [9];

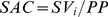
(6)

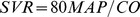
(7)where *SV_i_*, *PP, MAP, and CO* are stroke volume indexed by the body surface area, pulse pressure, mean arterial pressure and cardiac output, respectively.

1. Global (valvulo+arterial) LV load, estimated by the valvulo-arterial impedance (Z_VA_) [9], as follow:
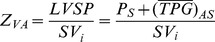
(8)where LVSP, 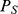 and 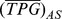 are LV systolic pressure, aorta systolic pressure and mean transvalvular pressure gradient, respectively

#### Cardiovascular Magnetic Resonance (CMR)

The CMR study was performed 2 to 4 weeks after the TTE study with the use of a 1.5 Tesla scanner and a dedicated cardiac phase-array receiver coil (Achieva, Philips Medical Systems, Best, The Netherlands). CMR image acquisitions and analyses were performed by investigators blinded to clinical and TTE results, as previously described [25], [26], [27].

A custom-made research application was developed using Matlab software (Mathworks, Natick, Ma) to process and analyze CMR images [24], [25], [26]. The valve *EOA* from CMR (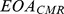) was then calculated as follow:
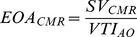
(9)where 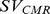 is the stroke volume using 1/3 Simpson’s rule to integrate the systolic flow and 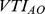 is the velocity-time integral of the peak aortic flow velocity measured at 10 mm downstream of the valve during systole [25], [27], [28], [29].

#### Computed normalized stroke work index (N-SW)

**Figure 2:**
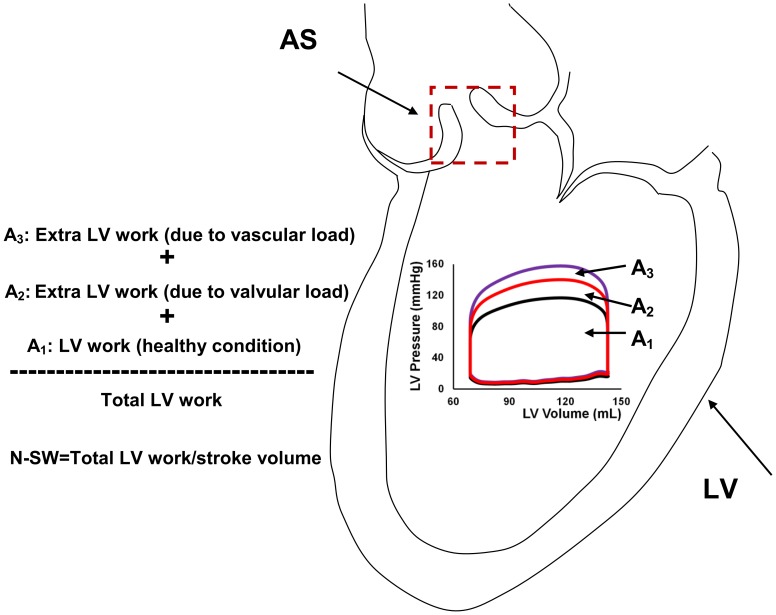
Schematic diagram. LV stroke work and N-SW.

We have introduced a lumped-parameter method for clinical practice that accurately investigates the impact of AS and concomitant vascular diseases on the LV function. This method only needs few non-invasively measured quantities described as follows. TTE and CMR can both provide the effective orifice area (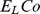) and aortic cross sectional area (*A*). Equation 4 uses these quantities to calculate the valvular energy loss coefficient (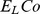). Subsequently 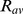 (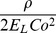) and 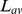 (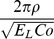) in Equation 3 and Fig. 1 are calculated. The total systemic resistance is computed as the average brachial pressure over the cardiac output and is used to calculate 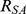 assuming constant values for other resistances in the circuit. The systemic compliance (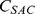) was adjusted by fitting the predicted pulse pressure to the measured pulse pressure. The LV stroke work, representing the work of the left ventricle during each heart beat (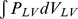), was then computed using the left ventricle volume, 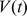, and the left ventricle pressure, 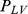. Normalized LV stroke work (N-SW) is calculated by dividing the estimated LV stroke work by the SV (see Figure 2 for schematic diagram).

#### Statistical analysis

Results were expressed as mean ± SD. CMR and TTE measurements were compared by 2-tailed paired Student t-tests or one-way ANOVA when appropriate. Association and agreement between variables were assessed by Pearson’s correlation and Bland-Altman analyses, respectively. Statistical analysis was performed with SPSS 17 (SPSS, Chicago, IL).

## Results

### Validation of the Lumped Parameter Model using *in vivo* CMR Flow Wave Forms

**Figure 3:**
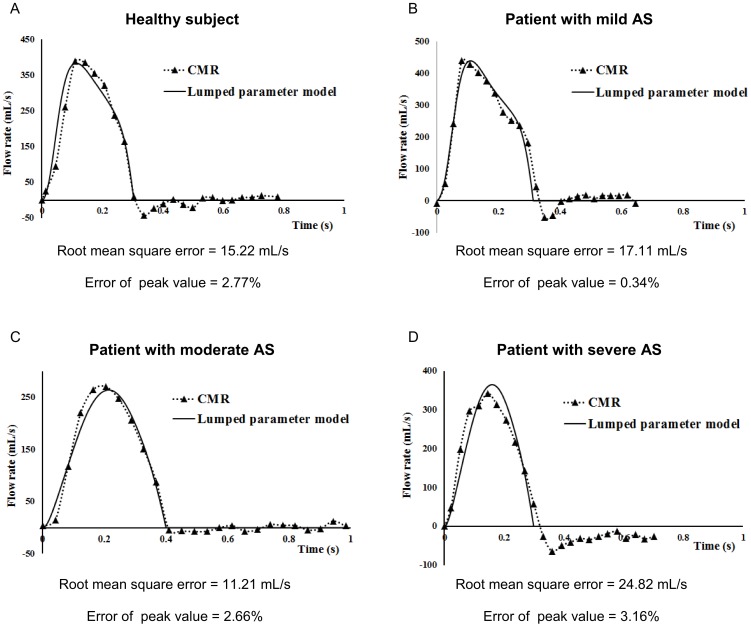
Comparison of simulated and CMR flow waveforms. (a) healthy subject, (b) patient with mild AS, (c) patient with moderate AS, (d) patient with sever AS.

Using the above mentioned protocol, data obtained non-invasively in forty-nine patients with mild to severe AS and eight healthy subjects (Table 2) were incorporated in the model and the LVOT flow waveforms resulted from the lumped parameter model were compared with *in vivo* CMR flow waveforms with an average root mean square error of 12.21 mL/s. There was a very good correlation between the peak values of flow waveforms obtained from simulations and CMR measurements (r = 0.98; SEE = 0.001, p<0.001). Figure 3 presents few examples of comparison between the simulated and CMR flow waveforms.

### Estimation of Normalized Stroke Work Based on the Input Data from TTE and CMR

**Figure 4:**
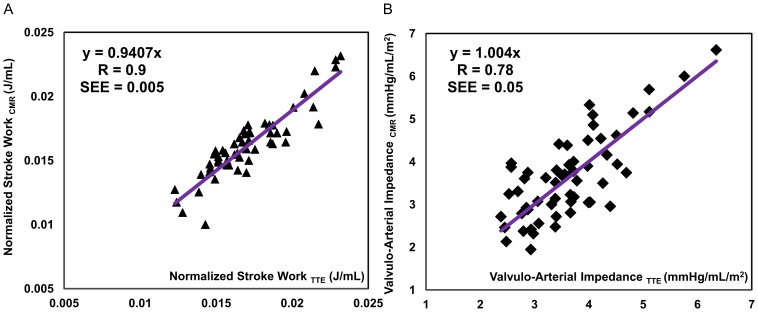
Correlation between results based on TTE and CMR measurements. (a) correlation between computed normalized left ventricular stroke works based on parameters measured by TTE and CMR, (b) correlation between computed valvulo-arterial impedance based on parameters measured by TTE and CMR.

Figure 4 shows a strong correlation between the results calculated using lumped parameter model based on TTE and CMR imaging datasets. Fig. 4a shows the correlation for the computed N-SW (r = 0.9, SEE = 0.005, p<0.001) and Fig. 4b shows the correlation for the *Z_VA_* (r = 0.78, SEE = 0.05, p<0.001).

### Correlation Analysis of the Normalized Stroke Work

**Figure 5:**
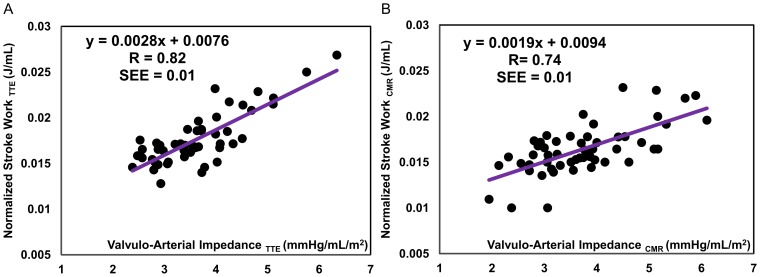
Correlation between normalized left ventricular stroke work and valvulo-arterial impedance. both computed using the lumped parameter model based on the parameters measured by (a) TTE and (b) CMR.

Figure 5 shows the strong correlation (TTE, Fig. 5a: r = 0.82, SEE = 0.01, p<0.001; CMR, Fig. 5b: r = 0.74, SEE = 0.01, p<0.001) between N-SW and 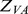, which has been shown to be a powerful predictor of mortality in patients with AS.

### Effect of the Flow Rate Condition on Normalized Stroke Work and Valvulo-arterial Impedance Based on TTE and CMR Input Data

**Figure 6:**
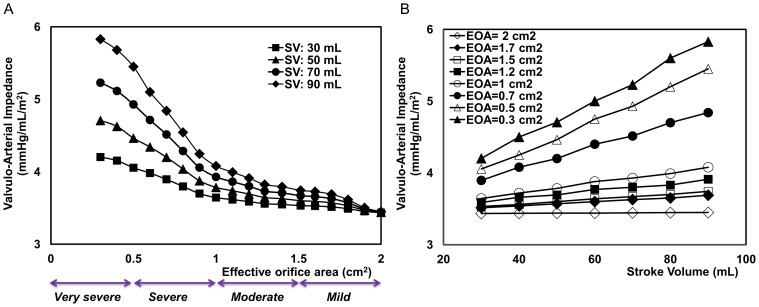
Dependency of valvulo-arterial impedance to effective orifice area and stroke volume. (a) as a function of effective orifice area (EOA), (b) as a function of stroke volume for normal arterial pressure.

**Figure 7:**
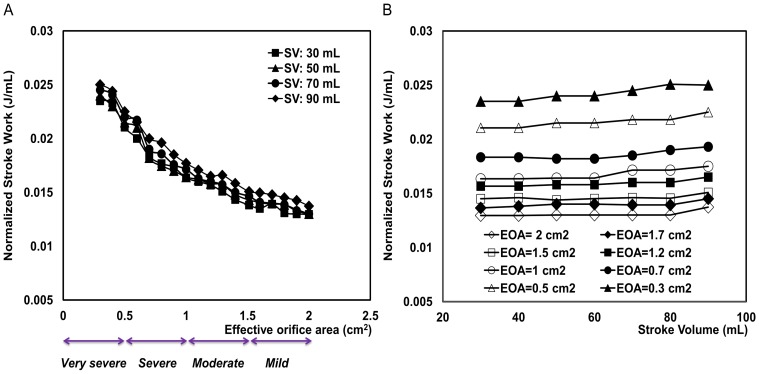
Dependency of normalized left ventricular stroke work to effective orifice area and stroke volume. (a) as a function of effective orifice area (EOA), (b) as a function of stroke volume for normal arterial pressure.

Figures 6 and 7 show the effect of the flow rate condition on 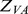 and N-SW. These figures illustrate hypothetical cases for a large range of stroke volumes: from 30 and 90 mL and with different AS severities: EOA of 0.3 to 2 cm^2^. Figure 6 demonstrates that 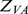 is significantly influenced by variations in flow rates: 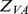 decreased when the stroke volume was reduced from 90 to 30 mL. In contrast, as Figure 7 shows, the flow rate had minimal effects on N-SW.

## Discussion

### Advantages of Normalized Stroke Work

Patients with AS often have concomitant arterial diseases. This should alert clinicians that the degenerative process is not only limited to the aortic valve, but also involves the arterial system distal to the valve. The latter can further contribute to deteriorating LV function [2], [30]. Under this condition, the LV faces an increased double load: a valvular load imposed by the AS plus an arterial load. Therefore, it is important to assess the hemodynamic load imposed on the LV. To this effect, 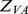 representing the valvular and arterial impedances opposing ventricular ejection, was recently proposed [4].

N-SW represents the global hemodynamic load that the LV faces for ejecting a unit volume of the blood. It has a very good correlation with 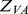. However, using N-SW to assess the AS has privilege to 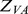 for two main reasons: 1) N-SW is not flow dependent while, by nature, 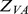 is. This may limit the utility of 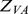 in patients with low-flow and low-gradient AS [8], 2) N-SW determines the actual total mechanical load imposed to the LV, while 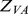 gives an estimate of that load.

### Estimation of Normalized Stroke Work and Valvulo-arterial Impedance by TTE versus CMR

This study demonstrates a very good correlation between the N-SWs computed based on the data from TTE and CMR methods. Similarly 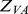 computed based on the data from TTE and CMR methods exhibit a very good correlation. TTE is the primary imaging technique for the assessment of AS severity and its progression. When TTE measurements are inconclusive or show discordances, AS severity should be confirmed with other techniques either invasively using cardiac catheterization or non-invasively using CMR [26], [31]. Results of this study suggest that there is a good agreement between TTE and CMR for the calculation of N-SW.

### Clinical Implications

Since the management of patients with AS remains a source of debate [3], [5], [6], [7], supplementing currently available indices with the N-SW could be useful in identifying patients who are at high risks and are in need of closer follow-ups and further investigations.

**Figure 8:**
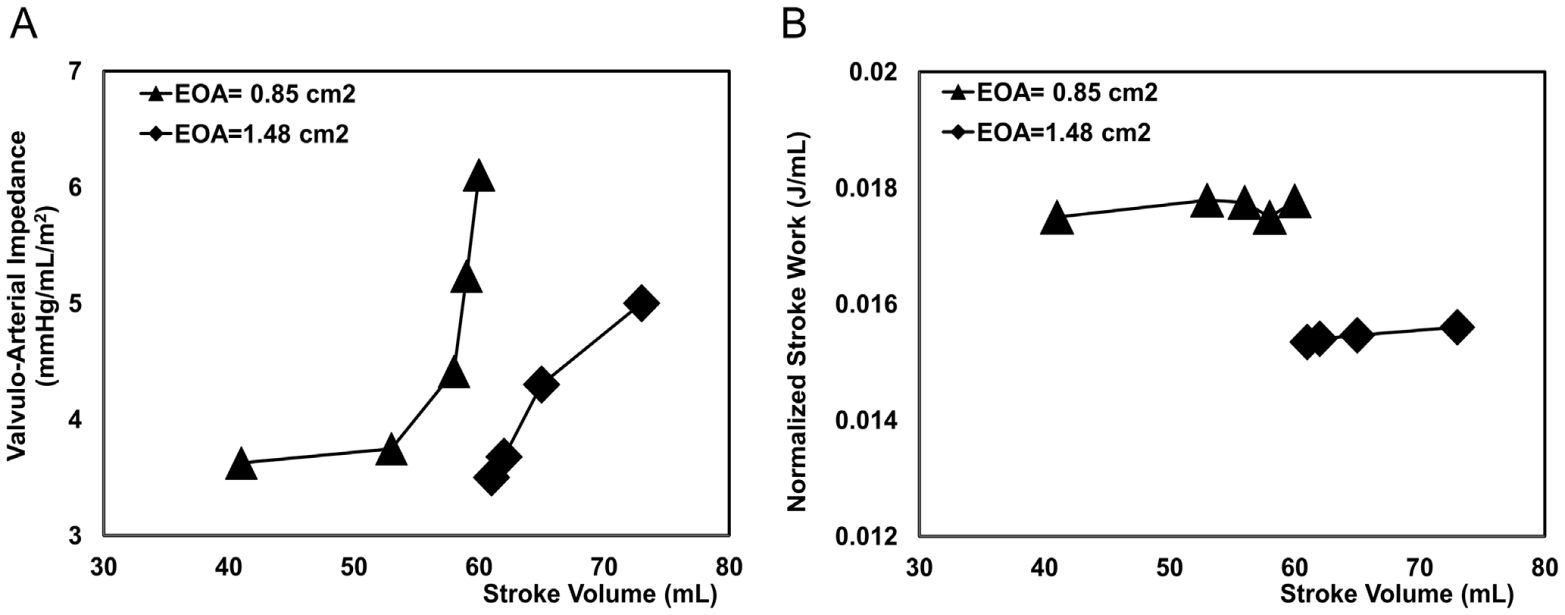
Valvulo-arterial impedance and normalized left ventricular stroke work. (a) Valvulo-arterial impedance (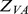) as a function of stroke volume, (b) normalized left ventricular stroke work (N-SW) as a function of stroke volume. In both panels a subset of patients with low flow and low pressure gradient conditions (Table 2) were considered. In these patients, AS was either moderate (EOA = 1.48 cm^2^, n = 4) or severe (EOA = 0.85 cm^2^, n = 5). In both groups, the stroke volume index (SVi) was less than 35 ml/m^2^ and the transvalvular pressure gradient was less than 20 mmHg. Both 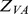 and N-SW were computed using the lumped parameter model based on the parameters measured by CMR.

More specifically, as already reported in [6], a significant number of patients with severe AS have low transvalvular flow rates (SVi <35 ml/m^2^), low transvalvular pressure gradients (<40 mmHg) and preserved LV ejection fractions (>50%). Clinically, this highly insidious situation represents a challenging clinical entity because the AS may appear less severe on the basis of low transvalvular pressure gradients. This situation may lead clinicians to erroneously conclude that no surgery is required because the stenosis is not considered to be severe. Indeed, these patients are at a more advanced stage of the disease. When compared with patients with severe AS but normal LV output, thus high pressure gradients, these patients are characterized by higher degrees of LV concentric remodeling, lower LVEFs and reduced mid-wall shortenings. It was found that these patients have poor prognoses and outcomes [6], [7]. Therefore, it can be conceived that a longstanding increase in the LV load may result in a more pronounced LV concentric remodeling, a smaller LV cavity size, and a decrease in intrinsic myocardial function. Early detection and accurate estimation of AS severity are, therefore, of primary importance. In this regard, for a comprehensive evaluation of the valvular and vascular loads, calculation of 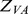 appears to be particularly useful for patients with AS. However, in low-flow low-gradient AS patients, 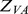 would likely be less precise in the assessment of the LV global hemodynamic load, due to its high flow-dependency (Figure 8a) [8]. In these patients, N-SW which is less flow-dependent, may be useful to better quantify the LV hemodynamic load and to better identify the actual disease severity that is masked by this paradoxical low flow phenomenon (Figure 8b).

## Conclusions

Patients with AS often have concomitant arterial diseases. Under this condition, the LV faces a double load: a valvular load imposed by the AS plus an arterial load. The double load results in increasing of the LV stroke work. We, therefore, introduced a lumped parameter method, capable of accurately assessing the impact of AS and concomitant vascular diseases on the LV workload using the data obtained non-invasively by TTE or CMR. The proposed method was then validated by comparing its estimated flow rates with *in vivo* CMR measurements.

We introduced a new index of LV hemodynamic load, the N-SW, representing the energy required to eject 1 mL of blood through the valvulo-arterial load. N-SW is less flow dependent than 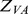. Findings of this study suggest that beyond standard indexes of stenosis severity and LV geometry and function, N-SW may be useful in order to improve risk stratification and clinical decision making in patients with AS, specifically in a subset of patients with low flow and low gradient conditions.
